# Incidence and Surgery Rate of Idiopathic Scoliosis: A Nationwide Database Study

**DOI:** 10.3390/ijerph18158152

**Published:** 2021-08-01

**Authors:** Sahyun Sung, Hyun-Wook Chae, Hye Sun Lee, Sinae Kim, Ji-Won Kwon, Soo-Bin Lee, Seong-Hwan Moon, Hwan-Mo Lee, Byung Ho Lee

**Affiliations:** 1 Department of Orthopedic Surgery, Ewha Womans University College of Medicine, Seoul 07804, Korea; sahyunsung@ewha.ac.kr; 2 Department of Pediatrics, Yonsei University College of Medicine, Seoul 03722, Korea; hopechae@yuhs.ac; 3 Biostatistics Collaboration Unit, Yonsei University College of Medicine, Seoul 03722, Korea; Hslee1@yuhs.ac (H.S.L.); ksn1214@yuhs.ac (S.K.); 4 Department of Orthopedic Surgery, Yonsei University College of Medicine, Seoul 03722, Korea; kwonjjanng@yuhs.ac (J.-W.K.); shmoon@yuhs.ac (S.-H.M.); hwanlee@yuhs.ac (H.-M.L.); 5 Department of Orthopedic Surgery, Catholic-Kwandong University, Incheon 22711, Korea; sumanzzz@ish.ac.kr

**Keywords:** Idiopathic scoliosis, incidence, medical utilization patterns, surgical treatment, nationwide database

## Abstract

Idiopathic scoliosis is the most common cause of three-dimensional deformities of the spine. Most of the previous studies have been cross-sectional studies to estimate the prevalence in the general population. An age-matched, population-based study is performed using nationwide databases between 2011 and 2015. The incidence rates of idiopathic scoliosis by age group, sex, and region are identified. We also investigate the pattern of medical institution use and the surgery rate of patients with idiopathic scoliosis. Our results show that a total of 268,372 patients were diagnosed with idiopathic scoliosis. The overall incidence was 0.497%, and the incidence for females was 1.44 times higher than for males. By age group, the incidence of adolescent idiopathic scoliosis in patients aged 10–14 years was 0.821% compared to 0.029%, 0.192%, and 0.709% for those patients aged 0–2, 3–9, and 15–19 years, respectively. Both male and female urban populations had higher incidences than rural populations with no age differences at diagnosis. Survival analysis confirmed that 0.7% of diagnosed patients underwent surgical treatment within five years. Understanding the epidemiology of idiopathic scoliosis is helpful in diagnosing high risk patients and monitoring surgical interventions.

## 1. Introduction

Idiopathic scoliosis is the most common cause of pediatric spinal deformities, comprising 80% of all scoliosis. Recently, idiopathic scoliosis was classified into four subcategories according to age of onset: Infantile, juvenile, adolescent, and adult [1]. Although, as the name implies, the etiology of idiopathic scoliosis is still unknown, it is believed that multifactorial causes, including biological, mechanical, hormonal, and genetic factors, contribute to its onset and progression [2,3,4,5,6].

The prevalence of adolescent idiopathic scoliosis (AIS) with a Cobb angle above 10° has been reported to vary from 0.93 to 12% worldwide [7,8,9,10,11,12,13,14]. Typically, AIS is more prevalent in females, and females tend to progress more than males [15,16]. The prevalence of infantile idiopathic scoliosis (IIS) and juvenile idiopathic scoliosis (JIS) is known to be much lower than that of AIS, and sex distributions show a different tendency from AIS [17].

Most of the previous idiopathic scoliosis studies were cross-sectional studies using a school-based screening program to estimate the prevalence in the general population. However, there have been no studies of when and how patients with idiopathic scoliosis were diagnosed and treated.

Therefore, the purpose of this study was to identify the incidence of idiopathic scoliosis and surgery rate using nationwide databases. 

## 2. Materials and Methods

This retrospective, longitudinal, epidemiologic study reviewed the nationwide cohort data between 2010 and 2015. This study was approved by the Institutional Review Board of the authors’ hospital. Informed consent of the patients was waived, since the study involved retrospective access to anonymized and publicly available data. 

### 2.1. Sources of Data and Data Selection

**Figure 1:**
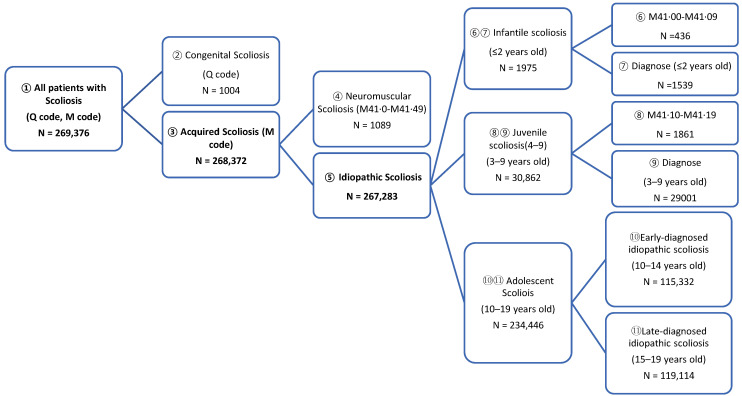
The diagram shows the nationwide scoliosis investigation from The Health Insurance Review and Assessment Service data analysis.

The universal health coverage system of South Korea, the National Health Insurance (NHI), covers the entire population. [18] The Health Insurance Review and Assessment (HIRA) Service is a quasi-governmental agency that evaluates whether insurance claims are appropriate. The claims data that HIRA collects includes extensive information, including not only performed procedures and prescribed medications, but also diagnoses, comorbidities, and demographic data of the insured patients. Insurance claims data of patients who were newly diagnosed with idiopathic scoliosis from 1 January 2011 to 31 December 2015 were collected. Diagnostic codes defined by the tenth revision of the International Classification of Diseases were used to identify patients with newly diagnosed idiopathic scoliosis (Figure 1). Those with diagnostic codes for congenital or neuromuscular scoliosis were excluded. The date of idiopathic scoliosis diagnosis was the date of the first idiopathic scoliosis indicator claim occurring between 1 January 2011 to 31 December 2015. Newly diagnosed idiopathic scoliosis beneficiaries had to have no idiopathic scoliosis indicator claims from 1 January 2010 until the date of their initial diagnosis. The diagnostic codes were registered by licensed doctors in medical institutions. 

### 2.2. Incidence of Idiopathic Scoliosis

The incidences of idiopathic scoliosis were analyzed in comparison to age-matched normal populations using data obtained from the government statistics office. The incidence rates were also analyzed according to the age classifications of idiopathic scoliosis: IIS, under 3 years; JIS, between 3 and 10 years; and AIS, between 10 and 19 years. AIS was further subdivided into ages 10–14 (early AIS) and 15–19 years (late AIS). Differences in incidences were further analyzed using urban and rural regions. The capital city, the metropolitan area around the capital, and six metropolitan cities were defined as urban areas; other provinces were defined as rural areas. Table 1 shows a comparison of basic information between urban and rural areas for 2016.

### 2.3. Surgical Treatment and the Disease Patterns of Idiopathic Scoliosis

Using HIRA data, we could identify the type of medical institutions where the patients were diagnosed, underwent surgery, and made follow-up visits. The medical institution in which the patient’s diagnosis code was first registered was considered the diagnostic institution, and the medical institutions at which the posterior-anterior X-ray scans of the whole spine (G4901, G4902, G4903, G4904, and G4905) were last taken were regarded as the last follow-up institutions. Operations were confirmed by registering the procedural codes (N0444, N0445, N0446, and N0447) between 2011 and 2015. Patients who satisfy the following criteria can be covered by the national insurance when they receive scoliosis surgery; (1) more than 40 degrees curvature in patients under 15 years of age, or (2) more than 50 degrees curvature in patients 15 years or older, or (3) scoliosis with thoracic lordosis.

The classification of medical institutions followed the criteria of the Ministry of Health and Welfare in Korea. Clinics are primary care institutions that have fewer than 30 beds. Hospitals have more than 30 beds and general hospitals, providing secondary care for inpatients, have more than 100 beds. Tertiary hospitals are nationally designated institutions among general hospitals that conduct difficult medical treatments for severe diseases. Institutions, such as nursing hospitals and oriental medical clinics, were classified as “Other”.

### 2.4. Statistical Analysis

Incidences were calculated as a rate per 100,000 population. A chi-squared test was performed to compare the incidence of idiopathic scoliosis according to sex and age. Survival analysis, calculated to determine how many and when the newly diagnosed idiopathic scoliosis patients underwent surgery, was performed using the Kaplan-Meier method. *p*-values < 0.05 were considered statistically significant. All statistical analyses were performed using SAS version 9.3 (SAS Institute Inc., Cary, NC, USA).

## 3. Results 

**Figure 2:**
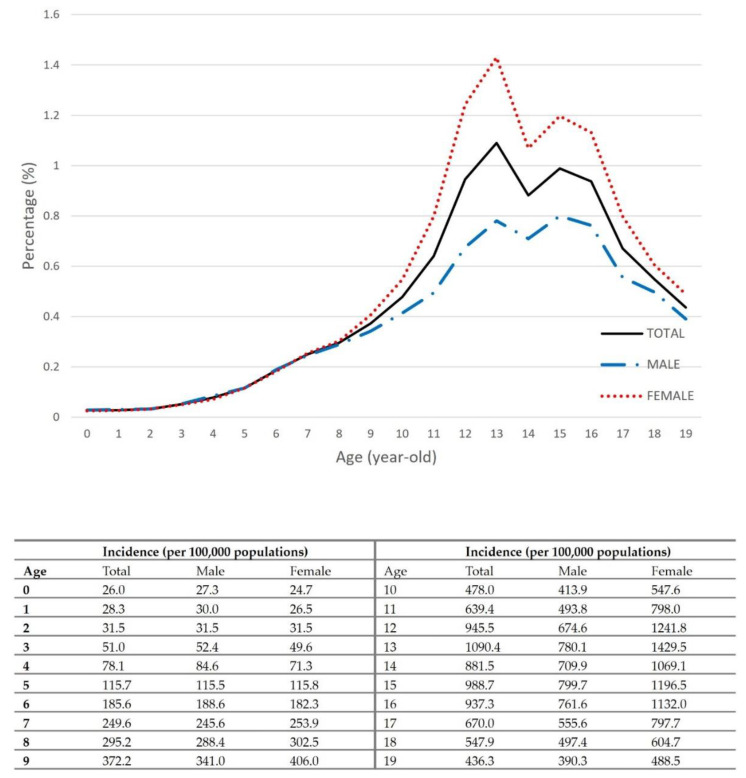
Age-specific incidence of idiopathic scoliosis between 2011–2015.

After excluding congenital and neuromuscular scoliosis, the number of patients diagnosed with scoliosis between 2011 and 2015 was 267,283. The overall incidence was 497 per 100,000 population, and the incidence of females was 1.44 times higher than that of males (*p* < 0.0001). Figure 2 shows the age-specific incidence rates for both males and females. Females had the highest incidence of 1430 per 100,000 population at the age of 13 years, and males had the highest rate at the age of 15 years (800/100,000 population). The first visit mean age was 13.56 ± 3.35 years: males, 13.54 ± 3.75; females, 13.64 ± 3.36 (*p* < 0.0001). The first visit mean age of AIS in females was 0.24 years younger than that of male (males, 14.69 ± 2.54; females, 14.45 ± 2.45 (*p* < 0.0001)). 

The incidence of IIS was 29 per 100,000 population—30 in males; 28 in females, with no statistical differences between the two groups (*p* = 0.1182). The overall incidence of JIS was 192 per 100,000 population—188 in males; 197 in females, which was 1.05 times higher than males (*p* < 0.0001). The overall incidence of AIS was 760 per 100,000 population, with the incidence of females (928/100,000 population) being 1.52 times higher than that of males (609/100,000 population) (*p* < 0.0001). The overall incidence of early AIS (10–14 years) was 821 per 100,000 population, with the incidence of females (1036/100,000 population) being 1.65 times higher than that of males (624/100,000 population) (*p* < 0.0001). The overall incidence of late AIS was 709 per 100,000 population, with the incidence of females (837/100,000 population) being 1.40 times higher than that of males (595/100,000 population) (*p* < 0.0001) (Table 2).

According to the analysis of regional incidence, urban groups showed higher incidences than rural groups in all age groups (Table 3). The urban population male incidence (445/100,000 population) was 1.34 times higher than that of rural inhabitants (333 /100,000 population) (*p* < 0.0001); the urban female population (625/100,000 population) was 1.22 times higher than the female rural population (511/100,000 population) (*p* < 0.0001). In particular, there was a large regional difference in the 3–9 year age group, with urban groups having 1.92 times and 1.83 times higher incidences in males and females, respectively. In males, the mean age at diagnosis for the urban group was 14.67 ± 2.57 years, and that of the rural group was 14.74 ± 2.44 years, while in females it was 14.43 ± 2.49 and 14.50 ± 2.32 years, respectively. 

We also identified the types of medical institutions where idiopathic scoliosis patients were diagnosed, underwent operations, and had follow-up visits (Table 4). The diagnosis of idiopathic scoliosis occurred mostly in clinics (54.10%), followed by hospitals (25.34%), general hospitals (9.49%), tertiary hospitals (5.69%), and other institutions (4.88%). Surgical treatment was most frequently performed in tertiary hospitals (87.34%), followed by general hospitals (7.07%), and hospitals (5.59%). The diagnosed patients were last followed up mostly at clinics (50.66%), followed by hospitals (25.28%), general hospitals (9.62%), tertiary hospitals (8.56%), and other institutions (5.88%).

**Figure 3:**
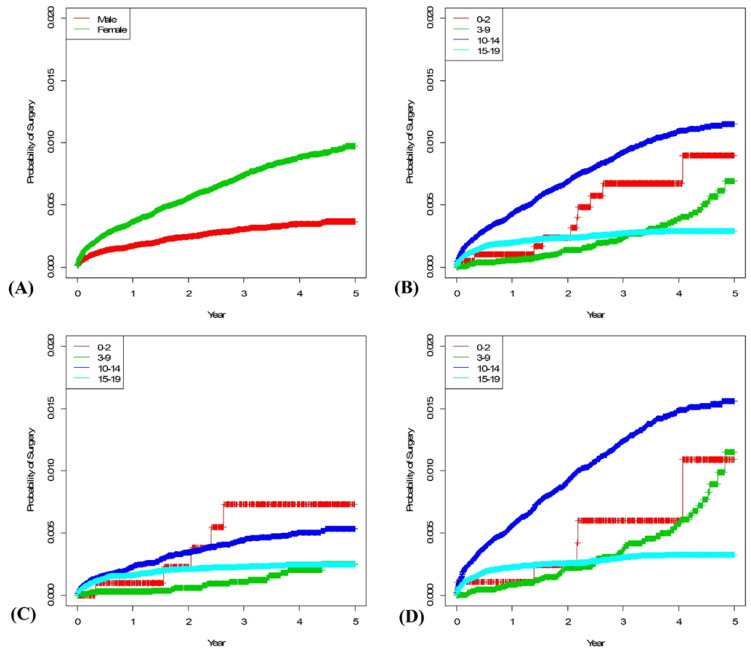
Each graph shows the Kaplan-Meier survival curve of surgery rate of idiopathic scoliosis patients: (**A**) Male vs. female, (**B**) by age group, (**C**) by age group in the male population, and (**D**) by age group in the female population.

A total of 1278 surgeries were performed during the 5-year period, of which 980 cases (76.68%) were performed in females. Ninety-seven percent of patients underwent surgery between the ages of 10 and 19 years. Seventy-one percent of female patients undergoing surgery were 10–14 years old, and 26% were 15–19 years old. Among the male patients undergoing surgery, 50% were 10–14 years old, and 45% were 15–19 years old. The 5-year survival rates were calculated according to sex and age, and the mean follow-up period was 4.80 years. The overall 5-year survival rate was 0.7% (newly diagnosed patients with idiopathic scoliosis underwent surgery within five years): males, 0.36%; females, 1.00%. The 5-year survival rate of IIS was 0.89%: males, 0.73%; females, 1.09%. Of those diagnosed with JIS, 0.69% underwent surgery: males, 0.25%; females 1.14%. Among AIS patients diagnosed at 10–14 years, 1.14% of patients underwent surgery within five years: males, 0.53%; females 1.54%. In AIS patients diagnosed at 15–19 years, 0.29% of patients underwent surgery within five years: males, 0.24%; females, 0.32% (Figure 3). 

## 4. Discussion

Epidemiological studies on the prevalence of idiopathic scoliosis have been conducted worldwide. In 1978, Rogala et al. [19] reported a prevalence rate of 2% between the ages of 12 and 14 years in a Montreal study, and Lonstein et al. [20], in 1982, reported a prevalence rate of 1.1% in Minnesota, USA. To date, most studies have been conducted by estimating the overall prevalence using a screening program within a specific population such as in schools, although this is controversial [21,22,23]. 

We were able to evaluate nearly the entire population because most of the South Korean population is insured by the NHI, and any claim data are sent to the HIRA for review. 

In addition, diseases such as scoliosis are unlikely to be underdiagnosed, because it is a relatively specific disease with precise diagnostic criteria. Korean patients have easy access to specialists in primary care, and the diagnostic codes are registered only by licensed doctors; thus, the registered diagnostic codes are unlikely to include misdiagnosed cases or those undergoing screening. Additionally, medical examinations are mandatory for children at the ages of 7, 10, 13, and 16 years [24]. The mandatory school screening program consists of a physical examination for scoliosis. When there are suspicious findings in the physical examination or chest X-ray, students are referred to the medical institutions for further evaluation. 

Since incidence rates were expected to vary greatly around the growth spurt, AIS was further divided into two groups: early (10–14 years) and late (15–19 years). The incidence was higher in the early group (821 per 100,000 population)—624 in males; 1036 in females. The overall incidence of the late group was 709 per 100,000 population; 595 in males; 837 in females. Suh et al. [12] reported the prevalence of AIS to be 3.26% (3260 per 100,000 individuals) in a study of South Korean patients aged 10–14 years. Since the prevalence includes all previously diagnosed patients and the incidence includes only newly diagnosed patients, this might explain why the incidence identified in this study was lower than in previous studies. 

The incidence was highest in 15 year-old males and 13 year-old females (Figure 2). The difference in peak ages between males and females may be due to the growth spurt periods. We anticipated that the onset or progression of scoliosis may be affected by the time of the growth spurt; however, the incidences of those aged 10–14 years (821/100,000 population) and those aged 15–19 years (709/100,000 population) were not very different. There is a possibility that patients older than the mean age of peak height growth can be newly diagnosed with scoliosis; therefore, adolescents over 15 years of age may still require monitoring. 

Although there was a difference in the incidence between males and females, the mean age at first visit did not differ too much between males and females (males, 14.69 ± 2.54; females, 14.45 ± 2.45 (*p* < 0.0001)). Many patients are likely to be diagnosed at the age when the mandatory screening is performed, and therefore, the incidence rate is likely to be higher in these age groups. There are few IIS and JIS epidemiologic studies because screening programs are rarely available at this age. When considering all ages of idiopathic scoliosis, the proportion of IIS was 1%, and that of JIS was approximately 10–20%. [25] In this study, the incidence of IIS was 29 per 100,000 population, which was very low compared to that of AIS, with no statistical differences between sexes. The incidence of JIS was 192 per 100,000 population, and the incidence was 1.05 times higher in females. In particular, the difference between sexes was found to increase after the age of 7 (Figure 2). 

The incidence rate for urban populations was significantly higher than that for rural populations. A previous meta-analysis study showed that adolescents living in high-latitude regions have a higher prevalence of IS. [26] However, there has not yet been an epidemiologic study for IS comparing urban and rural areas. It is possible that urban populations showed higher incidences than rural populations because of differences in accessibility to medical institutions, outdoor activity time, learning time, and sleeping time. Further studies are required to identify what characteristics of urban populations may lead to higher incidences of idiopathic scoliosis. It is unlikely that racial or latitude differences affected the outcomes, since the geographical latitudes did not vary greatly between the urban and rural areas, and the population of South Korea is not comprised of diverse racial backgrounds. 

Initial diagnoses were confirmed in various institutions, and the proportion of clinics (54.1%) and hospitals (25.34%) was particularly high. This is most likely because idiopathic scoliosis can be diagnosed through relatively simple modalities such as X-ray radiography. Most patients underwent surgeries at tertiary hospitals (87.34%), probably because the anterior, posterior, and circumferential procedures, which are known as the mainstay of surgical treatment options for idiopathic scoliosis, require a longer operative time and have higher complication rates than other spinal surgeries. [27] Final follow-up was primarily performed at clinics and hospitals. The proportion of patients who made final follow-up visits at tertiary hospitals (9.62%) was higher than the percentage of those who were first diagnosed there (5.79%), possibly because the patients who received surgeries likely continued visiting the institutions where they had undergone their surgeries. Almost all surgeries were performed between the ages of 10 and 19 years, and the majority of the patients were females. In females, the number of patients undergoing surgery at 10–14 years of age was much higher than the male 15–19 age group having similar numbers of patients in these age groups.

This study found that the operation rate of AIS was 3.90 per 100,000 population. Tsirikos et al. [28] reported that 4.4~9.8 per 100,000 population received an operation for AIS. The lower surgery rate in this study could be due to ethnic differences or environmental influence.

The survival analysis was also interesting. Except for the IIS, which had a small sample, both male and female patients who were diagnosed at 10–14 years had the highest rates of surgery within five years of diagnosis. The slope of the survival curve for females aged 10–14 did not reach a plateau within five years when they reached the ages of 15–19 years (Figure 3). Lonstein et al. [29] claimed that the idiopathic scoliosis curve progressed well before the age of menarche 10 times more often in females than males. They also suggested that <20° curve in > Risser stage 3 does not require treatment because it has a <2% chance of progression. Cho et al. [30] reported the mean age at menarche of Korean girls as 13.10 ± 0.06 years, and most girls attain Risser stage 3 within two years after menarche. Therefore, it may be presumed that after five years of follow-up for the 10–14 years old group, there would be less need for surgical interventions. However, our results showed that patients diagnosed at 10–14 years of age continued to require surgery after the age of 15 years. In the case of JIS, the slope of the curve gradually increased over time. These results were particularly prominent in females, and it is likely that scoliosis progressed as the patients’ ages approached the growth spurt period. Survival curves of patients diagnosed at the age of 15–19 reached plateaus after 3 years in both males and females presumably because their growth was completed, and the scoliosis curves no longer progressed. 

### Limitation

There are several limitations to this study. First, since the diagnosis of idiopathic scoliosis was identified only by registered diagnostic codes, there is a risk for underestimations of the groups; however, under-diagnosis is unlikely because nearly the entire population is covered by medical insurance and regular screening programs are conducted for adolescents. Secondly, since the data on the Cobb angle of the patient’s scoliosis curve were not included, the incidence of severe scoliosis and the outcomes according to curve angle could not be identified. Thirdly, the data used in this study included only those claimed by the HIRA, which review the insurance claims, and the incidence and outcomes of uninsured procedures, such as bracing treatments, could not be investigated. In addition, growth-sparing techniques, such as growing rods or vertical expandable prosthetic titanium ribs, do not have individual procedural codes and instead are included in the procedural code for posterior surgeries [31,32]. Therefore, if such procedures were performed, there could be an overestimation of the surgery rates, particularly in infants or juvenile patients. 

## 5. Conclusions

This study was the first to analyze the incidence of idiopathic scoliosis using a nationwide database. The incidence of IIS was 0.029%, JIS was 0.192%, and AIS was 0.760%. The incidence was highest among females between the ages of 10 and 14, and urban populations had higher incidences than rural populations. Most of the patients were diagnosed and followed up in primary care facilities, and most surgeries were performed in tertiary hospitals. Surgery was most frequently performed at 10–14 years of age. Understanding the epidemiology of idiopathic scoliosis would be helpful in monitoring patients at high risk of diagnosis or progression.

## Figures and Tables

**Table 1:** A comparison of basic information between urban and rural populations for 2016.

	Area (km^2^)	Population (1000 Persons)	Population Density (Persons/km^2^)	Physicians per 1000 Population
**Whole country**	100,339	51,218	510	2.74
**Urban**	16,071	35,696	2221	2.93
**Rural**	84,269	15,520	184	2.29

Urban: Capital city (Seoul), the metropolitan area around the capital (Gyeonggi-do) and six metropolitan cities (Incheon, Daejeon, Gwangju, Daegu, Ulsan, and Busan); Rural: Other provinces (Gangwon-do, Chungcheongbuk-do, Chungcheongnam-do, Jeollabuk-do, Jeollanam-do, Gyeongsangbuk-do, Gyeongsangnam-do, and Jeju-do).

**Table 2:** Annual incidences of idiopathic scoliosis by age groups.

Year	Total Population			Idiopathic Scoliosis Patients			Female to Male Ratio of Cases		Incidence (Per 100,000 Population)		
	Total	Male	Female	Total	Male	Female	Ratio (95% CI)	*p*-Value	Total	Male	Female
**Total**											
**2011**	11,239,996	5,881,170	5,358,826	64,136	27,514	36,622	1.458 (1.435–1.481)	<0.0001	571	468	683
**2012**	11,012,807	5,753,390	5,259,417	53,585	22,978	30,607	1.454 (1.430–1.480)	<0.0001	487	399	582
**2013**	10,765,287	5,611,677	5,153,610	51,453	21,880	29,573	1.469 (1.444–1.495)	<0.0001	478	390	574
**2014**	10,503,422	5,461,478	5,041,944	50,754	21,612	29,142	1.458 (1.433–1.484)	<0.0001	483	396	578
**2015**	10,252,151	5,317,005	4,935,146	47,355	21,177	26,178	1.330 (1.306–1.354)	<0.0001	462	398	530
**2011–2015**	53,773,663	28,024,720	25,748,943	267,283	115,161	152,122	1.435 (1.424–1.446)	<0.0001	497	411	591
**0–2 years old**											
**2011**	1,377,662	709,944	667,718	472	259	213	0.874 (0.729–1.048)	0.1465	34	36	32
**2012**	1,398,142	719,983	678,159	409	227	182	0.851 (0.700–1.035)	0.1051	29	32	27
**2013**	1,416,869	728,206	688,663	384	190	194	1.080 (0.884–1.319)	0.4525	27	26	28
**2014**	1,372,125	704,425	667,700	404	206	198	1.014 (0.834–1.232)	0.8887	29	29	30
**2015**	1,339,380	686,679	652,701	306	168	138	0.864 (0.690–1.082)	0.2036	23	24	21
**2011–2015**	6,904,178	3,549,237	3,354,941	1975	1050	925	0.932 (0.853–1.018)	0.1182	29	30	28
**3–9 years old**											
**2011**	3,274,307	1,698,130	1,576,177	7011	3566	3445	1.041 (0.993–1.091)	0.0943	214	210	219
**2012**	3,209,730	1,661,444	1,548,286	5887	2973	2914	1.052 (0.999–1.107)	0.0529	183	179	188
**2013**	3,172,280	1,639,689	1,532,591	5839	2912	2927	1.075 (1.022–1.132)	0.0055	184	178	191
**2014**	3,175,393	1,638,761	1,536,632	6046	3040	3006	1.054 (1.003–1.109)	0.0391	190	186	196
**2015**	3,201,817	1,650,662	1,551,155	6079	3113	2966	1.014 (0.964–1.066)	0.5909	190	189	191
**2011–2015**	16,033,527	8,288,686	7,744,841	30,862	15,604	15,258	1.046 (1.023–1.070)	<0.0001	192	188	197
**10–14 years old**											
**2011**	3,119,491	1,630,159	1,489,332	27,854	11,124	16,730	1.639 (1.600–1.679)	<0.0001	893	682	1123
**2012**	2,969,359	1,550,478	1,418,881	23,564	9383	14,181	1.645 (1.603–1.688)	<0.0001	794	605	999
**2013**	2,803,088	1,464,013	1,339,075	22,669	8967	13,702	1.664 (1.620–1.709)	<0.0001	809	612	1023
**2014**	2,666,175	1,389,826	1,276,349	21,871	8507	13,364	1.703 (1.658–1.750)	<0.0001	820	612	1047
**2015**	2,488,686	1,294,946	1,193,740	19,374	7787	11,587	1.608 (1.563–1.655)	<0.0001	778	601	971
**2011–2015**	14,046,799	7,329,422	6,717,377	115,332	45,768	69,564	1.652 (1.632–1.671)	<0.0001	821	624	1036
**15–19 years old**											
**2011**	3,468,536	1,842,937	1,625,599	28,799	12,565	16,234	1.460 (1.427–1.494)	<0.0001	830	682	999
**2012**	3,435,576	1,821,485	1,614,091	23,725	10,395	13,330	1.443 (1.407–1.481)	<0.0001	691	571	826
**2013**	3,373,050	1,779,769	1,593,281	22,561	9811	12,750	1.448 (1.411–1.487)	<0.0001	669	551	800
**2014**	3,289,729	1,728,466	1,561,263	22,433	9859	12,574	1.409 (1.372–1.446)	<0.0001	682	570	805
**2015**	3,222,268	1,684,718	1,537,550	21,596	10,109	11,487	1.243 (1.211–1.277)	<0.0001	670	600	747
**2011–2015**	16,789,159	8,857,375	7,931,784	119,114	52,739	66,375	1.402 (1.386–1.418)	<0.0001	709	595	837
**10–19 years old**											
**2011**	6,588,027	3,473,096	3,114,931	56,653	23,689	32,964	1.546 (1.520–1.572)	<0.0001	860	682	1058
**2012**	6,404,935	3,371,963	3,032,972	47,289	19,778	27,511	1.542 (1.514–1.570)	<0.0001	738	587	907
**2013**	6,176,138	3,243,782	2,932,356	45,230	18,778	26,452	1.553 (1.525–1.583)	<0.0001	732	579	902
**2014**	5,955,904	3,118,292	2,837,612	44,304	18,366	25,938	1.547 (1.518–1.576)	<0.0001	744	589	914
**2015**	5,710,954	2,979,664	2,731,290	40,970	17,896	23,074	1.403 (1.376–1.431)	<0.0001	717	601	845
**2011–2015**	30,835,958	16,186,797	14,649,161	234,446	98,507	135,939	1.520 (1.508–1.532)	<0.0001	760	609	928

**Table 3:** Regional incidences of idiopathic scoliosis in males and females.

	Total Population		Idiopathic Scoliosis Patients		Urban to Rural Ratio		Incidence (Per 100,000 Population)	
	Urban	Rural	Urban	Rural	Ratio (95% CI)	*p*-Value	Urban	Rural
**Male (years old)**								
**Total**	19,429,181	8,595,539	86,521	28,640	1.335 (1.317–1.353)	<0.0001	445	333
**0–2**	2,480,836	1,440,580	806	244	1.918 (1.662–2.213)	<0.0001	32	17
**3–9**	5,772,199	3,373,735	12,072	3532	1.996 (1.922–2.072)	<0.0001	209	105
**10–14**	5,058,018	2,271,404	34,618	11,150	1.392 (1.362–1.422)	<0.0001	684	491
**15–19**	6,118,128	2,739,247	39,025	13,714	1.272 (1.248–1.297)	<0.0001	638	501
**10–19**	11,176,146	5,010,651	73,643	24,864	1.326 (1.307–1.345)	<0.0001	659	496
**Female (years old)**								
**Total**	17,969,602	7,779,341	112,358	39,764	1.222 (1.208–1.236)	<0.0001	625	511
**0–2**	2,349,110	1,250,804	708	217	1.737 (1.492–2.022)	<0.0001	30	17
**3–9**	5,411,867	2,904,967	11,798	3460	1.829 (1.761–1.899)	<0.0001	218	119
**10–14**	4,655,069	2,062,308	51,510	18,054	1.261 (1.240–1.283)	<0.0001	1107	875
**15–19**	5,553,556	2,378,228	48,342	18,033	1.147 (1.127–1.166)	<0.0001	870	758
**10–19**	10,208,625	4,440,536	99,852	36,087	1.202 (1.187–1.216)	<0.0001	978	813

**Table 4:** Types of medical institutions where idiopathic scoliosis patients were first diagnosed, underwent surgery, and attended last follow-up visits.

Medical Institute	Initial Diagnosis			Surgery			Last Follow-Up Visit		
	Total (%)	Male (%)	Female (%)	Total (%)	Male (%)	Female (%)	Total (%)	Male (%)	Female (%)
**All**	267,283 (100%)	115,161 (100%)	152,122 (100%)	1288 (100%)	308 (100%)	980 (100%)	267,283 (100%)	115,161 (100%)	152,122 (100%)
**Tertiary hospital**	15,473 (5.79%)	6567 (5.7%)	8906 (5.85%)	1125 (87.34%)	266 (86.36%)	859 (87.65%)	22,881 (8.56%)	8612 (7.48%)	14,269 (9.38%)
**General hospital**	25,370 (9.49%)	10,523 (9.14%)	14,847 (9.76%)	91 (7.07%)	26 (8.44%)	65 (6.63%)	25,717 (9.62%)	10,731 (9.32%)	14,986 (9.85%)
**Hospital**	67,717 (25.34%)	29,213 (25.37%)	38,504 (25.31%)	72 (5.59%)	16 (5.19%)	56 (5.71%)	67,556 (25.28%)	29,239 (25.39%)	38,317 (25.19%)
**Clinic**	144,593 (54.1%)	62,472 (54.25%)	82,121 (53.98%)	0 (0%)	0 (0%)	0 (0%)	135,412 (50.66%)	59,661 (51.81%)	75,751 (49.8%)
**Others**	13,055 (4.88%)	5897 (5.12%)	7158 (4.71%)	0 (0%)	0 (0%)	0 (0%)	14,617 (5.47%)	6411 (5.57%)	8206 (5.39%)

## Data Availability

The data presented in this study are available on request from the corresponding author.
